# Successful treatment of pyopneumothorax secondary to *Streptococcus constellatus* infection with linezolid: a case report and review of the literature

**DOI:** 10.1186/s13256-020-02475-w

**Published:** 2020-10-07

**Authors:** Zhaorui Zhang, Binbin Xiao, Zhixin Liang

**Affiliations:** Department of Respiratory Medicine, Chinese PLA General Hospital, 28 Fuxing Road, Haidian District, Beijing City, 100853 People’s Republic of China

**Keywords:** Pyopneumothorax, *Streptococcus constellatus*, linezolid, case report, *Streptococcus milleri*, treatment

## Abstract

**Background:**

Pyopneumothorax secondary to *Streptococcus constellatus* infection is a clinically rare event, and few cases have been reported.

**Case presentation:**

We report the case of a 55-year-old Han Chinese man with underlying diabetes who presented with fever of 17 days duration. A pulmonary computed tomography scan revealed right-sided massive pyopneumothorax. A culture of the pleural effusion and blood grew *S. constellatus*. A drug sensitivity test showed that the isolate was sensitive to linezolid, penicillin G, cefotaxime, vancomycin, and cefuroxime. Our patient was treated with linezolid for a total of 6 weeks. Subsequently, his chest computed tomography scan showed improved lung condition.

**Conclusion:**

To the best of our knowledge, this is the first case of pyopneumothorax secondary to *S. constellatus* to be treated with linezolid. Pyopneumothorax may be caused by streptococcal infection, and linezolid is another good choice for treatment.

## Background

*Streptococcus constellatus* belongs to the *Streptococcus milleri* group of bacteria, which consists of *S. constellatus*, *Streptococcus intermedius*, and *Streptococcus anginosus* [1]. *S. constellatus* usually causes abscesses in various organs. Linezolid has not been reported to treat pyopneumothorax caused by *S. constellatus* infection. Here, we report a case of successful treatment of a patient with pyopneumothorax due to *S. constellatus* infection. This case contributes valuable information to the current knowledge on the treatment of this infectious disease.

## Case presentation

A 55-year-old Han Chinese man was admitted to our hospital on July 26, 2018 on presenting with a chief complaint of fever. Our patient began to have fever 17 days prior to his presentation, and his highest temperature had been 38.5 °C, accompanied by pain in his right chest and cough. He denied symptoms of chest tightness, nausea, and vomiting. He had taken oral amoxicillin without obvious effect, and the symptoms of fever and chest pain continued. He denied hepatitis, tuberculosis, and a history of hypertension or diabetes mellitus.

**Fig. 1:**
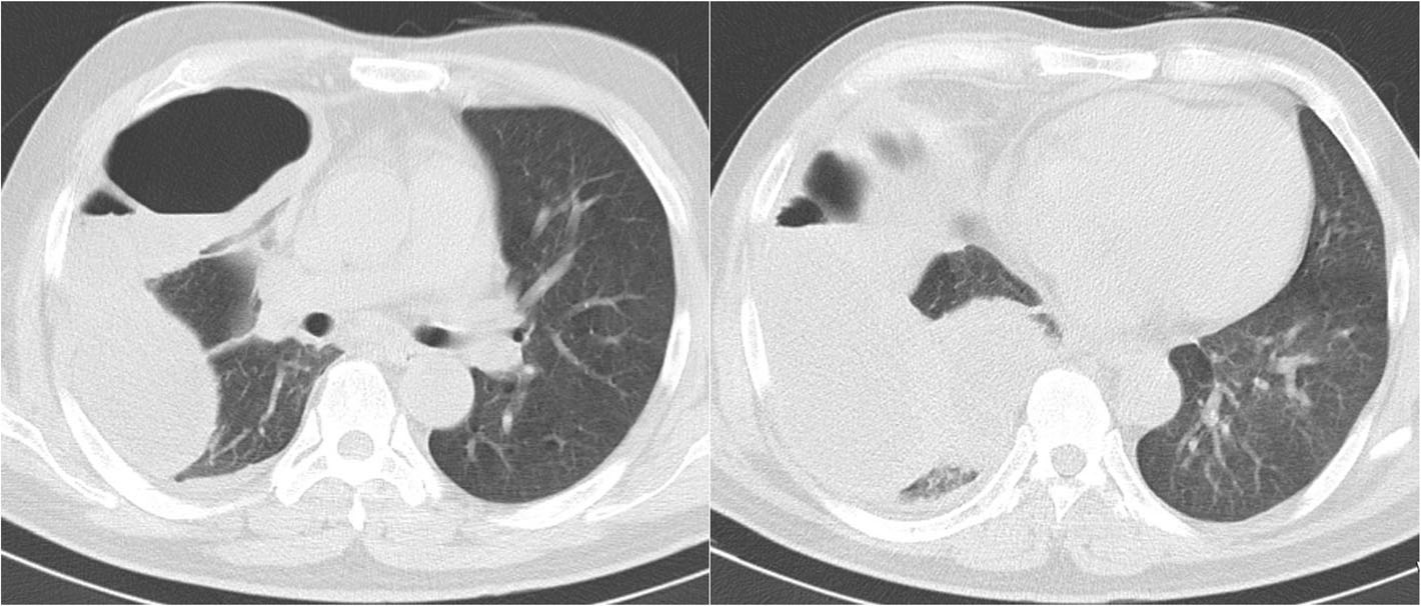
Chest computed tomography scan revealing right-sided massive pleural effusion with pneumothorax. Date: July 26, 2018

On admission, a physical examination revealed a temperature of 36.5 °C and blood pressure of 115/70 mmHg with a pulse rate of 75 beats per minute (bpm). A lung examination revealed reduced breath sound in the right lung field. The breath sound in the left lung was clear. A lung computed tomography (CT) scan (July 26, 2018) showed pyopneumothorax of the right lung and lower lobe right lung infection (Fig. 1); a cardiac ultrasound was normal.

### Investigation

Routine blood workup (July 26, 2018) results showed his WBC count was 19.24 × 10^9^/l(4–10 × 10^9^/l), neutrophils were 87.1% (50–70%), C-reactive protein was 10.6 mg/dl (0–0.8 mg/dl), serum albumin was 27.3 g/l (35–55 g/l), and his blood glucose was 11.06 mmol/l (3.9–6.1 mmol/l). Hepatitis B antigen and human immunodeficiency virus (HIV) antigen test results were all negative. His procalcitonin was 0.606 ng/ml (< 0.05 ng/ml), and glycosylated hemoglobin was 6.8% (4–6%). The initial diagnoses of our patient were pyopneumothorax, hypoalbuminemia, and type 2 diabetes. According to the local epidemiologic characteristics, differential diagnosis included lung abscess, community-acquired pneumonia, and pulmonary tuberculosis.

### Treatment

**Fig. 2:**
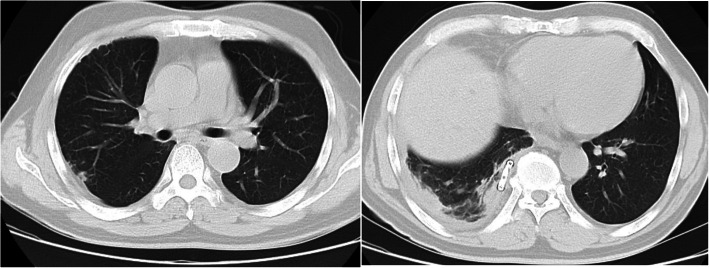
Chest computed tomography scan revealing right-sided pleural effusion and pulmonary infection. Date: October 11, 2018

Our patient was initially treated with closed chest drainage, and there was persistent drainage of pus from his right chest. He was treated with imipenem, linezolid to control the infection, and insulin to control his blood glucose. Pleural fluid culture and blood culture grew *S. constellatus*, which was sensitive to linezolid, penicillin G, cefotaxime, vancomycin, and cefuroxime. The drug susceptibility test is shown in Table 1. On October 8, 2018, the antibiotic regimen was changed to intravenous linezolid. There were no adverse or unanticipated events for linezolid treatment. On October 11, 2018, a chest CT scan (Fig. 2) showed reduced pleural effusion and pulmonary infection of the right lung. On October 24, 2018, the drainage tube was removed.

**Table 1:** Drug sensitivity to *Streptococcus milleri*

Drug	Blood culture		Pleural effusion culture	
	Sensitivity	Inhibition zone diameters (mm)	Sensitivity	Inhibition zone diameters (mm)
Penicillin G	Sensitive	29	Sensitive	29
Erythromycin	Resistant	6	Resistant	6
Clindamycin		18		19
Cefotaxime	Sensitive	26	Sensitive	25
Vancomycin	Sensitive	22	Sensitive	22
Linezolid	Sensitive	34	Sensitive	35
Cefuroxime	Sensitive	29	Sensitive	28

### Outcome and follow-up

**Fig. 3:**
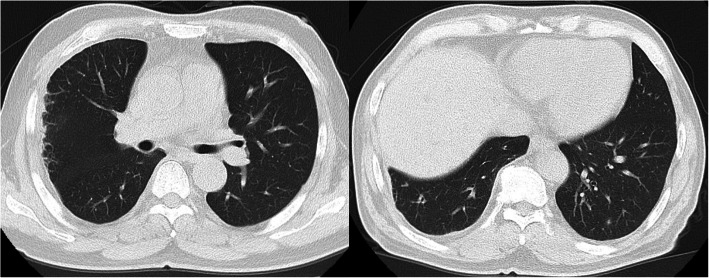
Chest computed tomography scan revealing slight right-sided pleural effusion and pulmonary infection. Date: November 16, 2018

Our patient was discharged with oral linezolid treatment for 1 week. He was prescribed a total of 6 weeks of linezolid treatment, including 5 weeks of intravenous linezolid and 1 week of oral linezolid treatment. A subsequent follow-up chest CT scan (Fig. 3) showed mass absorption of pyopneumothorax and pulmonary infection.

## Discussion and conclusion

Pyopneumothorax is the accumulation of gas and pus in the pleural cavity. Bacteria that frequently cause pyopneumothorax include *Pseudomonas aeruginosa*, *Escherichia coli* and *Streptococcus* [2].

The *S. milleri* group is a heterogeneous group of *Streptococci*, which is considered an important pathogen. It consists of three distinct species: *S. anginosis, S. constellatus and S. intermedius. S. milleri* has emerged in recent years as an organism associated with purulent disease in humans. *S. constellatus* is usually present in the normal flora of the mouth, vagina, respiratory tract, and gastrointestinal tract [3, 4]. Distinct species have been implicated in a number of pyogenic infections, including soft tissue infection, intra-abdominal and pulmonary abscesses, and central nervous system infections [5]. There is a considerable mortality rate from *S. milleri* infection. Fernando Cobo reported that the mean age at diagnosis was 62.06 ± 15 years. Two of 12 patients with *S. milleri* infection died as a consequence of infection. Risk factors for *S. milleri* infection include alcoholism, chronic obstructive pulmonary disease, and diabetes mellitus. The most frequently used antimicrobials for treatment were ceftriaxone and levofloxacin [5].

Cases of pyopneumothorax secondary to *S. milleri* infection have rarely been reported [6, 7]. Our case is the third case report of patients with pyopneumothorax caused by *S. milleri*. The summarization of the three cases are shown in Table 2. The first case was a 37-year-old man with hepatitis C infection. A chest radiograph and CT scan showed a large pleural cavity in the right hemithorax with an air-fluid level. The patient received empiric antibiotic therapy with cefuroxime and clindamycin. The patient recovered upon antibiotic treatment and drainage and was dismissed from the hospital after 17 days to home care [7]. The second patient was a 46-year-old Malay woman with underlying hypothyroidism post thyroidectomy who presented with worsening breathlessness, orthopnea, productive cough, and left-sided pleural chest pain of 3 days’ duration. The patient was treated with antibiotics for a total of 6 weeks and underwent open thoracotomy and decortication during admission [6]. In the current case, infection occurred primarily without any pre-existing pulmonary sequence or long treatment history. The patient was apparently healthy without any history of immunosuppression. However, her blood glucose was higher than normal, and diabetes was diagnosed on admission. The patient had undiagnosed type 2 diabetes before admission to our hospital. A previous study showed that diabetes is a risk factor for *S. milleri* group infection [5]; therefore, we deduced that the infection might have been related to the rise of blood glucose.

**Table 2:** Summarization of the three cases

	Age	Sex	Complication	Therapy
Case 1 [7]	37	Male	Hepatitis C	Cefuroxime and clindamycin
Case 2 [6]	46	Female	Hypothyroidism post thyroidectomy	Ceftazidime and metronidazole
Case 3	55	Male	Diabetes	Linezolid

In our case, we did not use cefuroxime and clindamycin as the empirical treatment. A drug sensitivity test showed that *S. milleri* was sensitive to linezolid, penicillin G, cefotaxime, vancomycin, and cefuroxime. Linezolid can be considered as the first member of the class of oxazolidinone antibiotics. Linezolid has been approved by the Food and Drug Administration for the treatment of the following: *Staphylococcus aureus*, *Streptococcus pneumonia*, and vancomycin-resistant *Enterococcus faecium* (VREF) infections [8]. A previous study also showed that *S. constellatus* isolated from liver abscesses was also sensitive to linezolid; however, the patient was treated with ceftriaxone [9]. The main side effects of linezolid were hematologic side effects including myelosuppression and thrombocytopenia. The ordinary dose of linezolid was 0.6 g per 12 hours. We had to monitor the blood routine for side effects. Our patient did not show any hematologic side effects and his platelet count was in the normal range.

Our case showed successful treatment of pyopneumothorax secondary to *S. constellatus* with linezolid for 6 weeks. Our case indicated that pyopneumothorax may be caused by streptococcal infection and that linezolid is a good choice for treatment.

## Data Availability

All data generated or analyzed during this study are included in this published article.

## Abbreviation

bpm: Beats per minute
HIV: Human immunodeficiency virus
VREF: Vancomycin-resistant Enterococcus faecium
WBC: White blood cell count
